# Host metabolic benefits of prebiotic exopolysaccharides produced by *Leuconostoc mesenteroides*

**DOI:** 10.1080/19490976.2022.2161271

**Published:** 2023-01-05

**Authors:** Junki Miyamoto, Hidenori Shimizu, Keiko Hisa, Chiaki Matsuzaki, Shinsuke Inuki, Yuna Ando, Akari Nishida, Ayano Izumi, Mayu Yamano, Chihiro Ushiroda, Junichiro Irie, Takane Katayama, Hiroaki Ohno, Hiroshi Itoh, Kenji Yamamoto, Ikuo Kimura

**Affiliations:** a Department of Applied Biological Science, Graduate School of Agriculture, Tokyo University of Agriculture and Technology, Fuchu-shi, Japan; b AMED-CREST, Japan Agency for Medical Research and Development, Chiyoda-ku, Japan; c Noster Inc. Kamiueno, Muko-shi, Kyoto, Japan; d Research Institute for Bioresources and Biotechnology, Ishikawa Prefectural University, Ishikawa, Japan; e Department of Bioorganic Medicinal Chemistry and Chemogenomics, Graduate School of Pharmaceutical Sciences, Kyoto University, Kyoto, Japan; f Laboratory of Molecular Neurobiology, Graduate School of Biostudies, Kyoto University, Kyoto, Japan; g Department of Molecular Neurobiology, Graduate School of Pharmaceutical Sciences, Kyoto University, Kyoto, Japan; h Department of Clinical Nutrition, Fujita Health University, Aichi, Japan; i Department of Endocrinology, Metabolism and Nephrology, School of Medicine, Keio University, Shinjuku-ku, Japan; j Laboratory of Molecular Biology and Bioresponse, Graduate School of Biostudies, Kyoto University, Kyoto, Japan; k Center for Innovative and Joint Research, Wakayama University, Wakayama, Japan

**Keywords:** Exopolysaccharides, short-chain fatty acids, dietary fiber, prebiotics, obesity, gut microbiota

## Abstract

Fermented foods demonstrate remarkable health benefits owing to probiotic bacteria or microproducts produced via bacterial fermentation. Fermented foods are produced by the fermentative action of several lactic acid bacteria, including *Leuconostoc mesenteroides*; however, the exact mechanism of action of these foods remains unclear. Here, we observed that prebiotics associated with *L. mesenteroides*-produced exopolysaccharides (EPS) demonstrate substantial host metabolic benefits. *L. mesenteroides*-produced EPS is an indigestible α-glucan, and intake of the purified form of EPS improved glucose metabolism and energy homeostasis through EPS-derived gut microbial short-chain fatty acids, and changed gut microbial composition. Our findings reveal an important mechanism that accounts for the effects of diet, prebiotics, and probiotics on energy homeostasis and suggests an approach for preventing lifestyle-related diseases by targeting bacterial EPS.

## Introduction

Fermented foods, which are produced through bacterial fermentation, have been an important food source for thousands of years. Fermented foods, such as yogurt, sauerkraut, kimchi, and pickles, involve the fermentation process by lactic acid bacteria. Consumption of these fermented foods provides us various health benefits. Fermented foods are rich in probiotic bacteria and enzymes to the gut and intestinal health microbiota, thereby maintaining overall health, digestive system health, and immune homeostasis.^1–4^ Moreover, prebiotics are present in fermentable dietary fiber such as insulin and β-glucan, and elicit beneficial health effects by changing the gut microbiota, because they pass undigested through the small intestine and stimulate the growth or activity of advantageous gut microbes in the colon. Therefore, interventions involving the combination of probiotics and prebiotics, also known as synbiotics, are important since they may exert beneficial health effects by improving the gut environment.

Lactic acid bacteria, such as *Leuconostoc mesenteroides*, are used in the fermentation process of fermented foods such as kimchi, sauerkraut, and pickles.^5,6^ *L. mesenteroides* is a gram-positive, spherical, facultative anaerobe^7,8^ that utilizes glucose, fructose, and sucrose as energy sources. *L. mesenteroides* converts sucrose to glucose and fructose via dextransucrase, a type of glycosyltransferase, and utilizes fructose as the energy source while producing dextran as an exopolysaccharide (EPS) from glucose.^7,9^ Dextran is a complex branched α-glucan polysaccharide of glucose.^7,9^ Notably, the main chain of *L. mesenteroides*-produced EPS is linked via α-1,6 glycosidic linkages between glucose monomers, with branches forming α-1,3 linkages.^7,10^ Presumably, *L. mesenteroides*-produced EPS (LmEPS) has an indigestible property as its glycosidic linkages cannot be broken by host amylase.^7,10^

The beneficial effects elicited by probiotic bacteria are not limited to changing the gut microbiota. Recent studies have demonstrated that various bacterial metabolites, particularly short-chain fatty acids (SCFAs), also exert beneficial effects on the human health.^11–14^ SCFAs, primarily acetate, propionate, and n-butyrate, are bacterial metabolites produced via complex carbohydrate fermentation. Gut microbes, such as *Bifidobacterium, Bacteroides*, and *Clostridium*, produce SCFAs from fermentable dietary fibers as polysaccharides, which are not absorbed into/in the small intestine because they are not digested by the host enzymes.^15^ Gut microbial SCFAs act as host energy sources and signal molecules via host receptors, such as the G protein-coupled receptors GPR41 and GPR43; they improve host homeostasis by impacting the endocrine systems.^16^ GPR41 affects host metabolic functions by increasing sympathetic activity and promoting the secretion of gut hormones,^16,17^ while GPR43 affects them by suppressing fat accumulation and promoting the secretion of gut hormones.^16,18^

The probiotic bacteria in fermented foods likely exert health benefits by changing the gut microbiota; however, the precise effects remain unclear. In the present study, we investigated the probiotic effects of *L. mesenteroides* by evaluating the role of LmEPS on host energy regulation and the molecular mechanism underlying the effect of microbial SCFAs.

## Methods

### Characterization of LmEPS

*L. mesenteroides* was cultured on MRS agar at 37°C or MRS agar containing 15% sucrose at 30°C for 48 h under anaerobic conditions and visualized using a scanning electron microscopy (SEM; JSM-7500 F; HUSRI, Aichi, Japan). LmEPS was extracted and purified as described previously,^19^ with certain modifications. Briefly, *L. mesenteroides* was cultured on MRS medium containing 15% sucrose at 30°C for 48 h under anaerobic conditions. After incubation, bacterial cells were removed via centrifugation. We precipitated the culture supernatant by adding an equal volume of chilled ethanol, mixing vigorously, and centrifuging at 8,000 g for 15 min. Subsequently, the supernatant was decanted. The precipitated LmEPS was dried over calcium chloride for 12–24 h. To determine its monosaccharide composition, LmEPS was hydrolyzed through the addition of trifluoroacetic acid (0.5 M) and incubated at 120°C for 2 h. After incubation, the supernatant was filtered through a 0.45 μm filter, and the monosaccharide composition was analyzed via ligand exchange chromatography using the 8.0 × 300 mm Shodex SUGAR SC1011 column (Shodex, Tokyo, Japan). Detection was performed using RID-10A (Shimadzu, Kyoto, Japan), and D-glucose and D-fructose (Nacalai Tesque, Kyoto, Japan) were used as standards. The average molecular mass of LmEPS was determined via size exclusion chromatography using the 8.0 × 300 mm Shodex OHpak SB-800 HQ series column (Shodex). Calibration curves for purchased average molecular weight 1,600,000, 788,000, 404,000, 212,000, 112,000, 47,300, 22,800, 11,800, 5,900 Pullulan (Shodex) and 1,500,000–2,800,000 dextrans (Sigma-Aldrich, St. Louis, MO, USA) were used as standards. In addition, the structure of LmEPS was confirmed using^1^H NMR spectroscopy. Briefly, LmEPS (1 mg) was dissolved in 750 μL of D_2_O containing 0.1% 3-(trimethylsilyl) propionic-2,2,3,3-d4 acid sodium salt as the chemical shift internal standard (δ_H_ 0.00 ppm). After allowing the solution to stand for 12 h, the^1^H NMR spectrum was recorded using the JEOL ECA-500 spectrometer at 500 MHz frequency (25°C). The residual H_2_O (in D_2_O) was used as an internal standard.

### Bacterial culture

Cultivation of *L. mesenteroides* in MRS medium containing 15% sucrose, 15% glucose, 15% fructose, and 7.5% glucose + 7.5% fructose was monitored for 24 h. The dominant gut bacteria were selected using a human gut microbial gene catalog^20^ and obtained from the Japan Collection of Microorganisms. Bacterial recovery was carried out according to the manufacturer’s instructions. The majority of bacteria were cultured on BL agar (Nissui, Tokyo, Japan), while *Lactobacillus* spp. were cultured on MRS agar at 37°C for 48 h under anaerobic conditions. Bacterial colonies on each medium were collected and mixed with nutrient broth (Difco Laboratories Inc.) containing 10% glycerol and stored at −80°C.

### RNA isolation and quantitative reverse transcriptase (qRT)-polymerase chain reaction (PCR)

Total RNA was extracted using RNeasy mini kit (Qiagen, Hilden, Germany), RNAiso Plus reagent (Takara Bio, Shiga, Japan), and NucleoSpin RNA kit (Takara Bio) and reverse transcribed to cDNA using Moloney murine leukemia virus reverse transcriptase (Invitrogen, Carlsbad, CA, USA). Subsequently, qRT-PCR analysis was performed using SYBR Premix Ex Taq II (Takara Bio) and the StepOnePlus real-time PCR system (Applied Biosystems), as described previously.^18^ The primer sequences were as follows: *putative glycosyltransferase-1*, 5′-ACAGCCGACAGTTGGTACAG-3′ (forward) and 5′-GCCTTATCTGGCCACCAAGT-3′ (reverse); *putative glycosyltransferase-2*, 5′-TCTTTAAGCGATCACCGGCA-3′ (forward) and 5′-TAACGATGGTGCCTTTGCCA-3′ (reverse); *putative dextransucrase-1*, 5′-ATGACAGCCCAAACGCAAAC-3′ (forward) and 5′-TCGTGTTCCAGCGTAAACTCA-3′ (reverse); *putative dextransucrase-2*, 5′-CCAAGTTGCTTTGCGGATGT-3′ (forward) and 5′-GATGGTGTTCGCCAAAGTGG-3′ (reverse); *putative dextransucrase-3*, 5′-AACGGATACGCAGCAGAACA-3′ (forward) and 5′-AGATTGCGTTGTGTCGCTTG-3′ (reverse); *aP2*, 5′-GATGCCTTTGTGGGAACCTGG-3′ (forward) and 5′-CTGTCGTCTGCGGTGATTTC-3′ (reverse); and *18S*, 5′-ACGCTGAGCCAGTCAGTGTA-3′ (forward) and 5′-CTTAGAGGGACAAGTGGCG-3′ (reverse).

### Genome sequencing

The complete genome of *L. mesenteroides* (strain NTM048) was constructed *de novo* using Illumina sequencing data. Sequencing analysis was performed by Chunlab, Inc. (300 bp × 2). Illumina data were assembled with SPAdes 3.10.1 (Algorithmic Biology Lab, St. Petersburg Academic University of the Russian Academy of Sciences). Resulting contigs were circularized using Circlator 1.4.0 (Sanger institute). To identify the adjacent gene involved in LmEPS biosynthesis, the gene-finding and functional annotation pipeline of whole genome assemblies in the EzBioCloud genome database was utilized. Protein-coding sequences (CDSs) were predicted using Prodigal 2.6.2.^21^ The CDSs were classified based on their roles, with reference to orthologous groups (EggNOG 4.5; http://eggnogdb.embl.de).^22^ All data analyses were performed using the Whole Genomics Database of EzBioCloud.

### SCFAs measurement

SCFAs in bacterial culture supernatants, feces, cecum, and plasma were determined as described previously,^23^ with certain modifications. The SCFA-containing ether layers were collected and pooled for gas chromatography-mass spectrometry (GC/MS) analysis using the GCMS-QP2010 Ultra GC mass spectrometer (Shimadzu). The calibration curves for the SCFAs were constructed, and their concentration in each sample was evaluated over a specified concentration range.

### Animals

C57BL/6 J, *Gpr41*^–*/*–^, *Gpr43*^–*/*–^, *Gpr41*^–*/*–^*Gpr43*^–*/*^^–^ double mutant, and ICR mice were housed under a 12-h light–dark cycle and given normal chow (CE-2; CLEA, Tokyo, Japan). Meanwhile, germ-free (GF)-ICR mice were housed in vinyl isolators under a 12-h light–dark cycle and given normal chow (CL-2, 50kGy irradiated; CLEA). *Gpr41*^–*/*–^, *Gpr43*^–*/*–^, and *Gpr41*^–*/*–^*Gpr43*^–*/*^^–^ double mutant mice were generated as described previously^17,18,24^ and were maintained on a C57BL/6 J genetic background. All experimental procedures involving mice were performed in accordance with the protocols approved by the Committee on the Ethics of Animal Experiments of the Kyoto University Animal Experimentation Committee (Lif-K21020) and the Tokyo University of Agriculture and Technology (permit number: 28–87).

For the experiment with orally administered LmEPS,^25^ each mouse was fed 0.2 g low-fiber diet (AIN-76A) containing 50% cellulose or 50% LmEPS for 1 h following a 24-h fasting. After feeding, the mice were euthanized, and the plasma and cecum were collected.

For high-fat diet (HFD) studies,^26^ 4-week-old C57BL/6 J and *Gpr41*^–*/*–^*Gpr43*^–*/*^^–^ double mutant mice were fed a D12492 diet (60% kcal fat; Research Diets) or modified D12492 diet for 12 weeks. Diet compositions are provided in Supplementary Table 1. Alternatively, 4-week-old C57BL/6 J mice were fed a *L. mesenteroides*-containing D12492 diet (Lm, 1 × 10^9^ cfu/g) for 12 weeks.

For gnotobiotic studies, male GF-ICR mice were fed a modified AIN-76A diet (Research Diets) for 2 weeks. Subsequently, each bacterial strain (1 × 10^8^ cfu/mouse) was administered via oral gavage thrice a week. Diet compositions are provided in Supplementary Table 3.

### Gut microbial composition

Fecal DNA was extracted from frozen samples using the FastDNA SPIN kit for feces (MP Biomedicals) as described previously.^27,28^ Partial 16S rRNA gene sequences were amplified by targeting the hypervariable regions v4 using the primers 515 F (5′-TCGTCGGCAGCGTCAGATGTGTATAAGAGACAGGTGYCAGCMGCCGCGGTAA-3′) and 806 R (5′-GTCTCGTGGGCTCGGAGATGTGTATAAGAGACAGGGACTACHVGGGTWTCTAAT-3)′. Amplicons generated from each sample were subsequently purified using AMPure XP (Beckman Coulter, Brea, CA, USA) and sequenced using the MiSeq sequencer (Illumina, San Diego, CA, USA) and MiSeq Reagent kit (version 3.0; 600 cycles). The 16S rRNA sequence data were then processed using the Quantitative Insights into Microbial Ecology (QIIME 1.8.0; http://qiime.org/) pipeline, and analyzed using the MiSeq Reporter software with the SILVA database (Illumina). Diversity analyses were performed using the QIIME script core_diversity_analyses.py. The statistical significance of sample groupings was assessed using a permutational multivariate analysis of variance (QIIME script compare_categories.py).

Furthermore, qPCR was performed using SYBR Premix Ex Taq II (Takara Bio) and the StepOnePlus real-time PCR system (Applied Biosystems). The primer sequences were as follows: *Muribaculum* spp., 5′-AGGGAGCAATTGAGTCCACG-3′ (forward) and 5′-TGATATTCCGCCTACGCACC-3′ (reverse); *Paramuribaculum* spp., 5′-TAATACGGAGGATGCGAGCG-3′ (forward) and 5′-CAAGGCACCCAGTTTCAACG-3′ (reverse); *Duncaniella* spp., 5′-TAATACGGAGGATGCGAGCG-3′ (forward) and 5′-GCATTCCGCATACTTCTCGC-3′ (reverse); *Akkermansia* spp., 5′-CAGCACGTGAAGGTGGGGAC-3′ (forward) and 5′-CCTTGCGGTTGGCTTCAGAT-3′ (reverse); *Bifidobacterium* spp., 5′-TCGCGTCYGGTGTGAAAG-3′ (forward) and 5′-CCACATCCAGCRTCCAC-3′ (reverse); *Faecalitalea* spp., 5′-GGGAACCCTGAACGAGCAAT-3′ (forward) and 5′-GCACGTAGTTAGCCGTGACT-3′ (reverse); *Blautia* spp., 5′-TCTGATGTGAAAGGCTGGGGCTTA-3′ (forward) and 5′-GGCTTAGCCACCCGACACCTA-3′ (reverse); *Streptococcus* spp., 5′-TGTCCGGATTTATTGGGCGT-3′ (forward) and 5′-ACTCTCCCCTTCTGCACTCA-3′ (reverse); *Muribaculum intestinale*, 5′-GTGGAAAATCGATCCGCTGC-3′ (forward) and 5′-ACCCCAGAGTCCGATGAGAA-3′ (reverse); *Paramuribaculum intestinale*, 5′-CAAACGTAAGCAGCGATCCG-3′ (forward) and 5′-ATCGCTGGGAGCTGATGATG-3′ (reverse); *Duncaniella muris*, 5′-AAGACGCCACGTGTGGCGTTCC-3′ (forward) and 5′-ACGGCTCGAAGAGGTTGAGCCTCTCA-3′ (reverse); *Bacteroides ovatus*, 5′-TGCAAACTRAAGATGGC-3′ (forward) and 5′-CAAACTAATGGAACGCATC-3′ (reverse); *Bacteroides stercoris*, 5′-GCTTGCTTTGATGGATGGC-3′ (forward) and 5′-CATGCGGGAAAACTATGCC-3′ (reverse); *Bacteroides thetaiotaomicron*, 5′-GCAAACTGGAGATGGCGA-3′ (forward) and 5′-AAGGTTTGGTGAGCCGTTA-3′ (reverse); *Akkermansia muciniphila*, 5′-CAGCACGTGAAGGTGGGGAC-3′ (forward) and 5′- CCTTGCGGTTGGCTTCAGAT-3′ (reverse); and 16S, 5′-ACTCCTACGGGAGGCAGCAGT-3′ (forward) and 5′-ATTACCGCGGCTGCTGGC −3′ (reverse).

### Histology

Adipose tissues were fixed in 10% neutral-buffered formalin, embedded in paraffin, and cut into 7 μm sections. Hematoxylin and eosin staining was performed, and the adipocyte area was measured as described previously.^27^

### Biochemical analyses

Blood glucose concentrations were measured using the OneTouch UltraVue glucometer (LifeScan, Milpitas, CA, USA) and LFS Quick Sensor (LifeScan). The concentrations of plasma NEFAs (LabAssay^TM^ NEFA; Wako Pure Chemical Co. Ltd., Osaka, Japan), triglycerides (LabAssay^TM^ Triglyceride; Wako Pure Chemical Co. Ltd.), total cholesterol (LabAssay^TM^ Cholesterol; Wako Pure Chemical Co. Ltd.), insulin (Mouse Insulin enzyme-linked immunosorbent assay [ELISA]; Shibayagi, Gunma, Japan), active glucagon like peptide-1 (GLP-1) (GLP-1 [Active] ELISA; Merck Millipore, Billerica, MA, USA), and peptide YY (PYY) (Mouse/Rat PYY ELISA Kit; Wako Pure Chemical Co. Ltd.) were measured according to the manufacturer’s instructions.

For GLP-1 measurement, the plasma samples were treated with a dipeptidyl peptidase IV inhibitor (Merck Millipore) to prevent degradation of active GLP-1. For the oral glucose tolerance test (OGTT), 16-h-fasted obese mice were administered glucose (2 g/kg of body weight) via oral gavage. For the insulin tolerance test (ITT), 3-h-fasted obese mice were intraperitoneally injected with human insulin (0.75 mU/g; Sigma-Aldrich). Plasma glucose concentration was monitored before and 15, 30, 60, 90, and 120 min after injection.

Following a 24-h fasting, 7-week-old C57BL/6 J, *Gpr41*^–*/*–^, *Gpr43*^–*/*–^, *Gpr41*^–*/*–^*Gpr43*^–*/*–^, ICR, and GF-ICR healthy male mice were fed 0.2 g AIN-76A containing 50% cellulose or 50% LmEPS.^25,29,30^ Alternatively, 7-week-old C57BL/6 J healthy male mice were administrated PBS or *L. mesenteroides* (1 × 10^9^ cfu/mouse) via oral gavage. After 1 h, each mouse was administered glucose (2 g/kg of body weight) via oral gavage or intraperitoneally. Plasma glucose was collected from the tail vein and measured using the OneTouch UltraVue glucometer and LFS Quick Sensor before and 15, 30, 60, 90, and 120 min after injection. Plasma samples were collected from the inferior vena cava at 15 or 30 min with the administration of glucose for insulin and GLP-1 measurement.

### Statistical analysis

All values are presented as the mean ± standard error of the mean. The normality of the data was assessed using the Shapiro–Wilk test (normal distribution was defined at *P* ≥ .05). We used the Student’s T-test to assess statistical significance between two groups following a normal distribution, and the Mann–Whitney U test was used compare groups in which the data did not follow a normal distribution. Data among multiple groups (≥ three groups) were compared using one-way analysis of variance (ANOVA), followed by the Dunnett’s post-hoc test or the Kruskal–Wallis test paired with the Dunn’s post-hoc test for normally versus non-normally distributed sample sets, respectively. Statistical significance was set at *P* < .05. Furthermore, false discovery rates (*Q*-value) of 16S rRNA gene sequencing data were estimated using the Benjamini–Hochberg procedure. Correlations between microbiota (bacterial genus abundances, *Muribaculum, Paramuribaculum, Duncaniella, Akkermansia, Bifidobacterium, Blautia, Faecalitalea*, and *Streptococcus*) and gut environmental factors (fecal SCFAs, such as acetate, propionate, and n-butyrate) were analyzed by calculating the Spearman’s rank correlation coefficient. Correlations with an absolute Spearman’s correlation coefficient above 0.6 with *Q*-value < 0.05 were selected. The Smirnov–Grubbs’ test was used for evaluating outliers.

## Results

### L. mesenteroides *produces EPS*

*L. mesenteroides* produced large amounts of EPS in MRS agar or broth containing 15% sucrose.^7,8^ In contrast, these bacteria did not produce EPS in MRS medium containing 15% glucose, 15% fructose, and 7.5% glucose + 7.5% fructose even though they proliferated (Supplementary Figure 1a).

Since dextran EPS is synthesized by glycosyltransferase^5^ and dextransucrase,^7^ we extracted two putative glycosyltransferase- and three putative dextransucrase-encoding genes from the draft genome sequences of *L. mesenteroides* (Supplementary Figure 2). The mRNA expressions of the putative glycosyltransferase-1, dextransucrase-2, and dextransucrase-3 genes were markedly increased along with the EPS yield in the medium containing 15% sucrose but not glucose (Supplementary Figure 1b). Hence, LmEPS may be synthesized by putative glycosyltransferase-1, dextransucrase-2, and dextransucrase-3. We then purified the LmEPS via ethanol precipitation and analyzed its composition via^1^H NMR spectroscopy in comparison with dextran.^31^ The purified LmEPS comprised dextran consisting of α-glucan, with main chain glucose monomers linked via α-1,6 glycosidic linkages and those present in branches linked via α-1,3 linkages (Supplementary Figure 1c).

## Gut microbial fermentation of LmEPS

**Figure 1.**
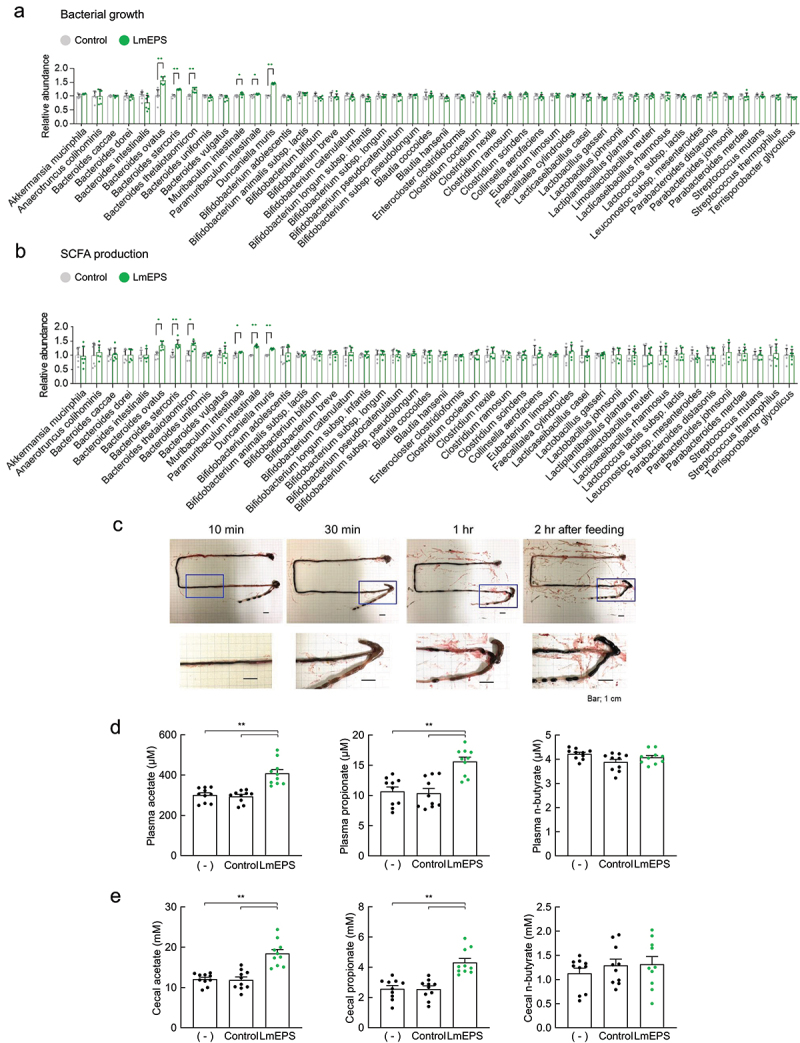
Short-chain fatty acids (SCFAs) produced from *Leuconostoc mesenteroides*-produced exopolysaccharide (LmEPS). (a) Bacterial growth and (b) SCFA production (acetate, propionate, and n-butyrate) in the culture supernatants of each gut bacteria (*n* = 6 per group). **P < .01, *P < .05 versus control (Mann–Whitney U test). (c) The intestinal position of the black dye-stained LmEPS after mice were fed an 0.2 g AIN-76A diet containing 50% LmEPS. (d) Plasma and (e) cecal SCFAs in mice fed an 0.2 g AIN-76A diet containing 50% cellulose (control) or 50% LmEPS for 1 h were measured using gas chromatography-mass spectrometry (*n* = 10 per group). **P < .01 (Dunn’s post-hoc test). Results are presented as the mean ± standard error of the mean (SE).

Next, we examined the bacteria related to SCFA production using *in vitro* gut microbial-monoculture screening. Among the 46 gut microbial strains evaluated, species belonging to the genus *Bacteroides*, such as *B. ovatus, B. stercoris*, and *B. thetaiotaomicron*; and *Bacteroidales* S24-7 group members, such as *Muribaculum intestinale, Paramuribaculum intestinale*, and *Duncaniella muris*, efficiently proliferated and produced SCFAs after addition of 0.3% LmEPS.^32^ However, other gut microbes, such as *Akkermansia, Bifidobacterium, Blautia, Clostridium, Lactobacillus*, and *Streptococcus*, did not produce SCFAs (Figure 1a, b). Furthermore, we examined the SCFA levels in the mouse intestine following intake of LmEPS. We first investigated the time for LmEPS to reach the cecum and colon by administrating LmEPS stained with black dye. As shown in Figure 1c, the black dye-stained LmEPS reached the cecum approximately 30 min and 1 h after administration. The levels of plasma and cecal SCFAs (acetate and propionate, but not n-butyrate) were markedly higher in low-fiber diet (LFD)-fed mice administered with LmEPS than those in LFD-fed mice and in those administered with cellulose (Figure 1d, e). Thus, LmEPS intake promoted SCFA production by gut microbes in the host intestine.

## Influence of LmEPS-derived gut microbial SCFAs on host glucose homeostasis

**Figure 2.**
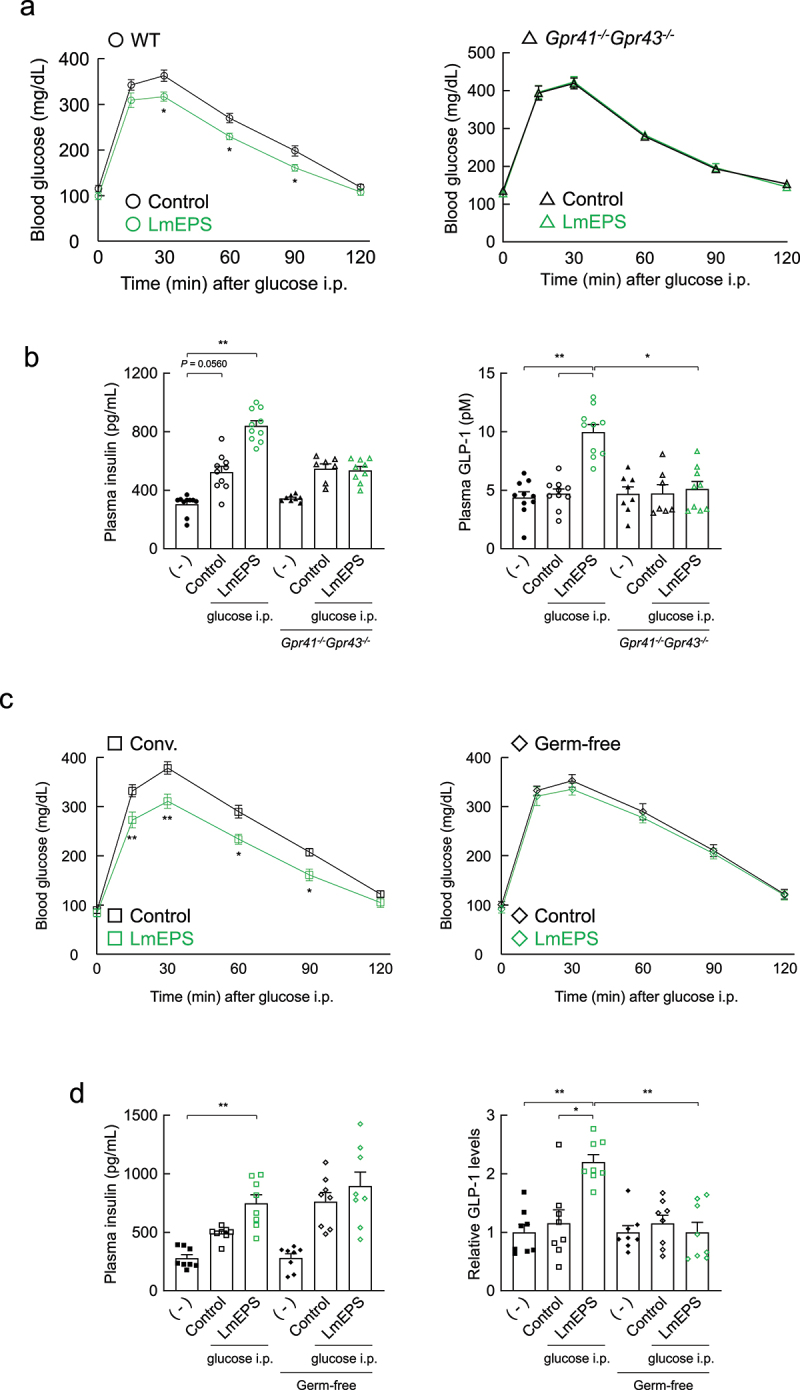
Effects of *Leuconostoc mesenteroides*-produced exopolysaccharide (LmEPS) on glucose homeostasis in mice. (a) After 24-h fasting, each mouse was fed a 0.2 g AIN-76A diet containing 50% cellulose (control) or 50% LmEPS, and an intraperitoneal glucose tolerance test was performed 1 h after feeding. Wild-type (*n* = 10 per group) and *Gpr41^–/–^Gpr43^–/^^–^* (*n* = 8 per group) C57BL/6 J mice were used. *P < .05 (Mann–Whitney U test). (b) The plasma insulin and glucagon like peptide-1 (GLP-1) levels were measured 15 min after intraperitoneal glucose administration. Wild-type (*n* = 10 per group) and *Gpr41^–/–^Gpr43^–/^^–^* (*n* = 8, 7, 9 per group) C57BL/6 J mice were used. **P < .01, *P < .05 (Dunn’s post-hoc test). (c) After 24-h fasting, each mouse was fed a 0.2 g AIN-76A diet containing 50% cellulose (control) or 50% LmEPS, and an intraperitoneal glucose tolerance test was performed 1 h after feeding. ICR (*n* = 8 per group) and germ-free (GF)-ICR mice were used (*n* = 8 per group). **P < .01, *P < .05 (Mann-Whitney U test). (d) The plasma insulin and GLP-1 levels were measured 15 min after intraperitoneal glucose administration. ICR (*n* = 8 per group) and GF-ICR mice (*n* = 8 per group) were used. **P < .01, *P < .05 (Dunn’s post-hoc test). Results are presented as means ± standard error of the mean (SE).

We examined the effects of LmEPS-derived gut microbial SCFAs on host glucose homeostasis. Dietary fiber-derived gut microbial SCFAs regulate the secretion of gut hormones, such as the incretin hormone GLP-1, via the SCFA receptors GPR41 and GPR43, thereby maintaining energy homeostasis and glucose metabolism.^13,16,33^ Therefore, we examined the effects of LmEPS on glucose homeostasis by performing a GTT. The administration of LmEPS significantly suppressed the increase in blood glucose level following glucose administration compared to that in control mice, whereas this effect was abolished in *Gpr41^–/–^Gpr43^–/–^, Gpr41^–/–^*, and *Gpr43^–/^^–^* mice (Figure 2a and Supplementary Figure 3a). Moreover, plasma insulin and incretin GLP-1 levels following glucose administration in LmEPS-administered mice were higher than those in control mice, and these effects were abolished in *Gpr41^–/–^Gpr43^–/^^–^* and *Gpr43^–/^^–^* but not in *Gpr41^–/^^–^* mice (Figure 2b and Supplementary Figure 3b). Additionally, under germ-free conditions, the LmEPS-mediated suppression of blood glucose increases and LmEPS-mediated increase in plasma insulin and GLP-1 levels were abolished (Figure 2c, d and Supplementary Figure 3c, d). In contrast, intake of *L. mesenteroides* did not exert any significant effect on glucose homeostasis (Supplementary Figure 4a). Thus, the intake of LmEPS rather than that of *L. mesenteroides*, improved host glucose homeostasis via the production of gut microbial SCFAs.

## Improved energy homeostasis following continuous intake of EPS

**Figure 3.**
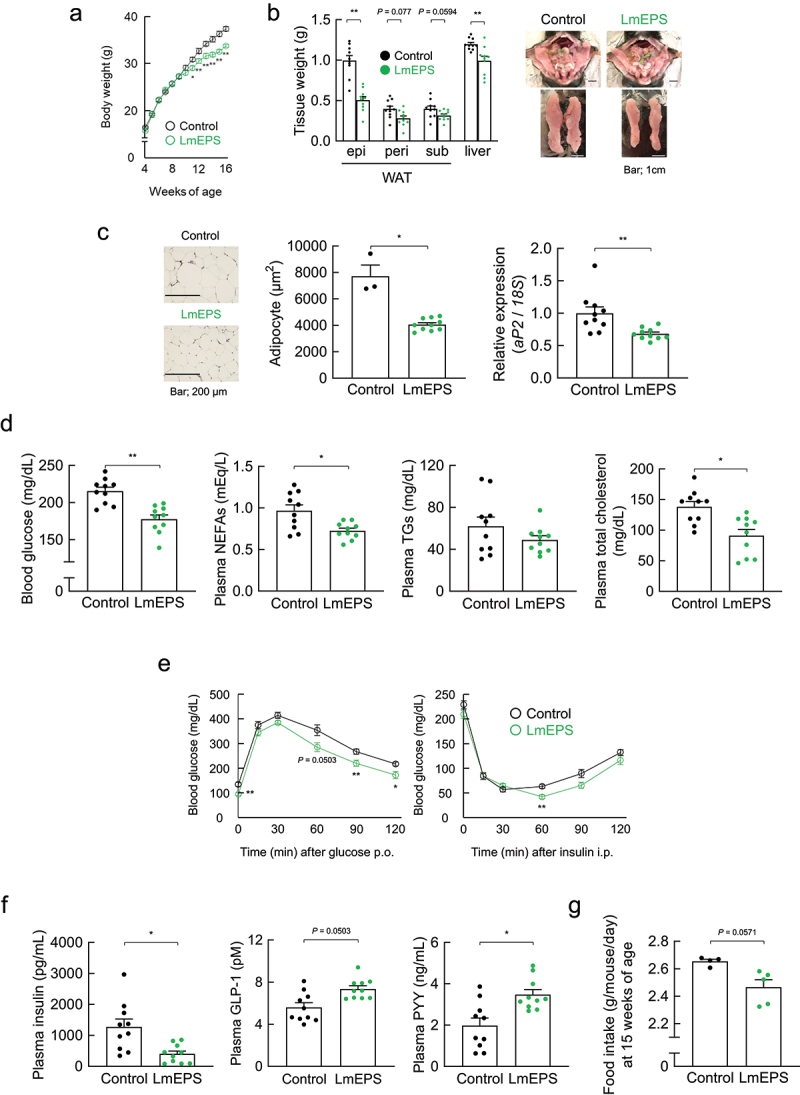
*Leuconostoc mesenteroides*-produced exopolysaccharide (LmEPS) improves metabolic condition in high-fat diet (HFD)-induced obesity. (a) Changes in body weight (*n* = 10 per group). (b) Changes in tissue weight and representative macroscopic appearance (*n* = 10 per group). Scale bar; 1 cm. epi, epididymal; peri, perirenal; sub, subcutaneous. (c) Hematoxylin and eosin–stained white adipose tissue (WAT) and the mean area of adipocytes (*n* = 3, 10 per group). Scale bar; 200 μm. *aP2* mRNA expression in the WAT of HFD-induced obese mice (*n* = 10 per group). (d) Blood glucose, plasma non-esterified fatty acids (NEFAs), plasma triglycerides (TGs), and plasma total cholesterol were measured at the end of the experimental period (*n* = 10 per group). Oral glucose tolerance test (Left; n = 10 per group) and insulin tolerance test (Right; n = 10 per group) were performed at 13–14 weeks of age. (f) Plasma insulin (n = 10 per group), glucagon like peptide-1 (GLP-1, n = 10 per group), and peptide YY (PYY, n = 10 per group) levels were measured at 16 weeks of age. (g) The daily food intake at 15 weeks of age (n = 4, 5 per group). Mice were fed an HFD containing cellulose (control) or LmEPS. **P< .01, *P <.05 (Mann–Whitney U test). Results are presented as the mean ± standard error of the mean (SE).

**Figure 4.**
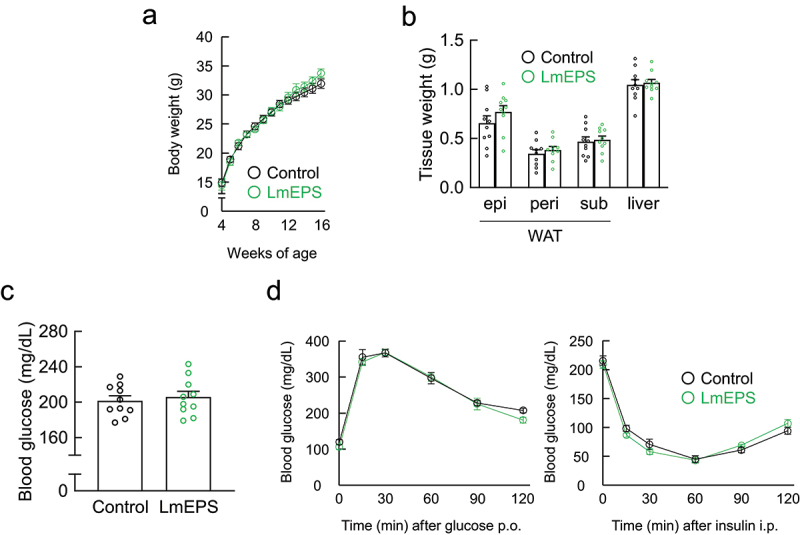
Beneficial metabolic effects of *Leuconostoc mesenteroides*-produced exopolysaccharide (LmEPS) were abolished in high-fat diet (HFD)-fed *Gpr41^–/–^Gpr43^–/^^–^* mice. (a) Changes in the body weights of *Gpr41^–/–^Gpr43^–/^^–^* mice (*n* = 10 per group). (b) Changes in tissue weights of *Gpr41^–/–^Gpr43^–/^^–^* mice (*n* = 10 per group). epi, epididymal; peri, perirenal; sub, subcutaneous. (c) Blood glucose was measured at the end of the experimental period (*n* = 10 per group). (d) Oral glucose tolerance test (*Left, n* = 8, 10 per group) and insulin tolerance test (*Right, n* = 8, 10 per group) were performed at 13–14 weeks of age. Mice were fed an HFD containing cellulose (control) or LmEPS. Results are presented as the mean ± standard error of the mean (SE).

We further examined the effects of LmEPS on host energy homeostasis in an HFD-induced obese mouse model. In this experiment, 4-week-old mice were fed a HFD supplemented with either LmEPS or cellulose as a nonfermented fiber for 12 weeks (Supplementary Table 1). The body weight of LmEPS-fed mice was markedly lower than that of cellulose-fed control mice during growth (Figure 3a). In addition, the fat mass of white adipose tissue (WAT) was significantly lower in LmEPS-fed mice than in control mice at 16 weeks of age (Figure 3b). A marked decrease in adipocyte size and mRNA levels of the adipose marker *aP2* were observed in the WAT of LmEPS-fed mice (Figure 3c). Furthermore, the plasma glucose, non-esterified fatty acids (NEFAs), and total cholesterol levels of LmEPS-fed mice were significantly lower than those of control mice, whereas the triglyceride level was comparable between the two groups (Figure 3d). HFD-induced insulin resistance and impaired glucose tolerance, as determined via the ITT and GTT, respectively, were significantly attenuated in LmEPS-fed mice compared to control mice (Figure 3e). In addition, the levels of plasma insulin were significantly lower and those of plasma GLP-1 and PYY were sufficiently higher in LmEPS-fed mice than those in control mice (Figure 3f). Furthermore, the resulting food intake in the LmEPS-fed mice tended to be lower than that in the control mice (Figure 3g). In contrast, these LmEPS-induced effects, such as suppression of body and adipose weight gain (Figure 4a,b), reduced hyperglycemia (Figure 4c), and improved insulin sensitivity (Figure 4d), were abolished in *Gpr41^–/–^Gpr43^–/^^–^* double mutant mice. Furthermore, compared with the intake of LmEPS, that of *L. mesenteroides* exerted less potent effects in terms of metabolic improvement (Supplementary Figures 4b–e). Thus, continuous intake of LmEPS improved energy homeostasis.

## Continuous intake of EPS alters the composition of the gut microbiota

**Figure 5.**
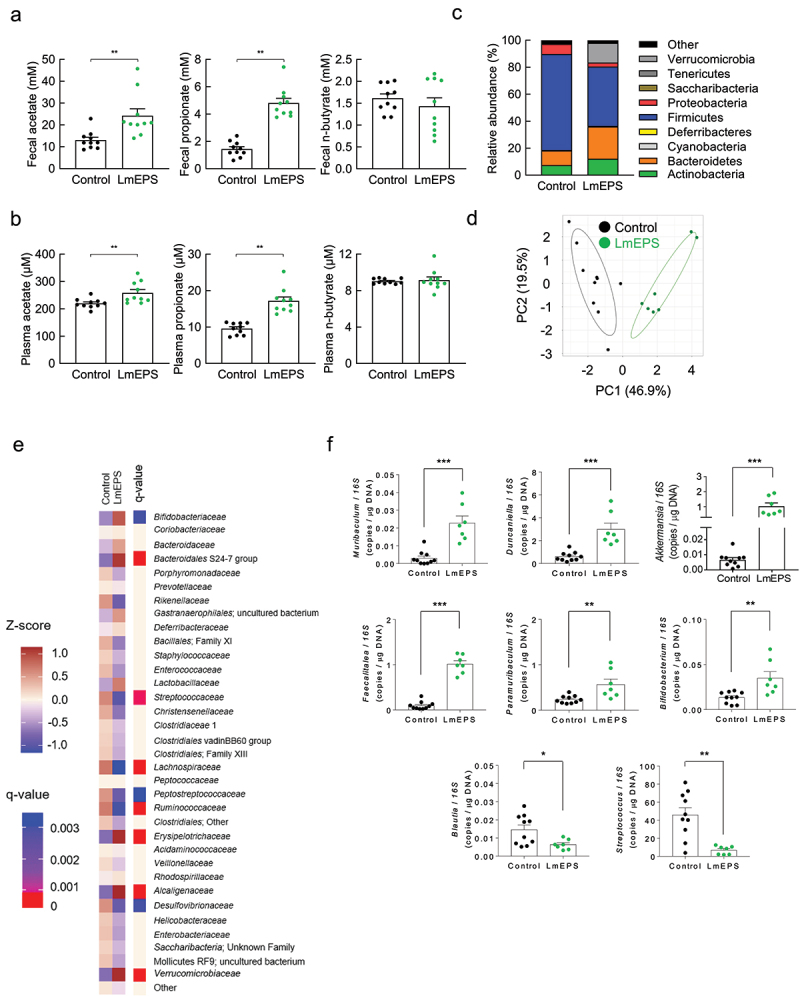
*Leuconostoc mesenteroides*-produced exopolysaccharide (LmEPS) improves the intestinal environments. (a) Fecal and (b) plasma short-chain fatty acids (SCFAs) were measured using gas chromatography-mass spectrometry (*n* = 10 per group). (c–f) Gut microbial composition was evaluated for the determination of relative abundance at the phylum level (c), principal coordinates analysis (d), heatmap of the bacterial domain at the family level (e), and at the genus level (f) (*n* = 10, 7 per group). Mice were fed a high-fat diet containing 10% cellulose (control) or 10% LmEPS. ***P < .001; **P < .01, *P < .05 (Mann–Whitney U test). Results are presented as the mean ± standard error of the mean (SE).

Continuous LmEPS intake markedly increased the levels of SCFAs, particularly propionate, in feces and plasma (Figure 5a, b). Therefore, we hypothesized that propionate plays an important role in host beneficial effects following the intake of LmEPS, and consequently, we assessed the LmEPS-mediated changes in gut microbial composition and investigated the propionate-producing gut microbes. Using 16S rRNA amplicon sequencing, we confirmed that supplementation with LmEPS altered the relative abundance of the major phyla that constitute the gut microbiota (Figure 5c). Specifically, the abundance of Bacteroidetes and Verrucomicrobia markedly increased, while that of Firmicutes markedly decreased in LmEPS-fed mice (Figure 5c). Hierarchical clustering of individual families confirmed the effect of LmEPS on the gut microbiome (Figure 5d). In contrast, although intake of *L. mesenteroides* tended to alter the gut microbial composition, it did not have any major effect on SCFA production (Supplementary Figure 5a–d). Changes in the gut microbiota following LmEPS intake were associated with the abundance of several gut microbe families (Figure 5e). Thus, we compared these gut microbes at the genus level (Figure 5f) and performed a correlation analysis between gut microbes and SCFAs.

## *Bacteroidales* S24-7 group or *Bacteroides* produce SCFAs from EPS in the intestine

**Figure 6.**
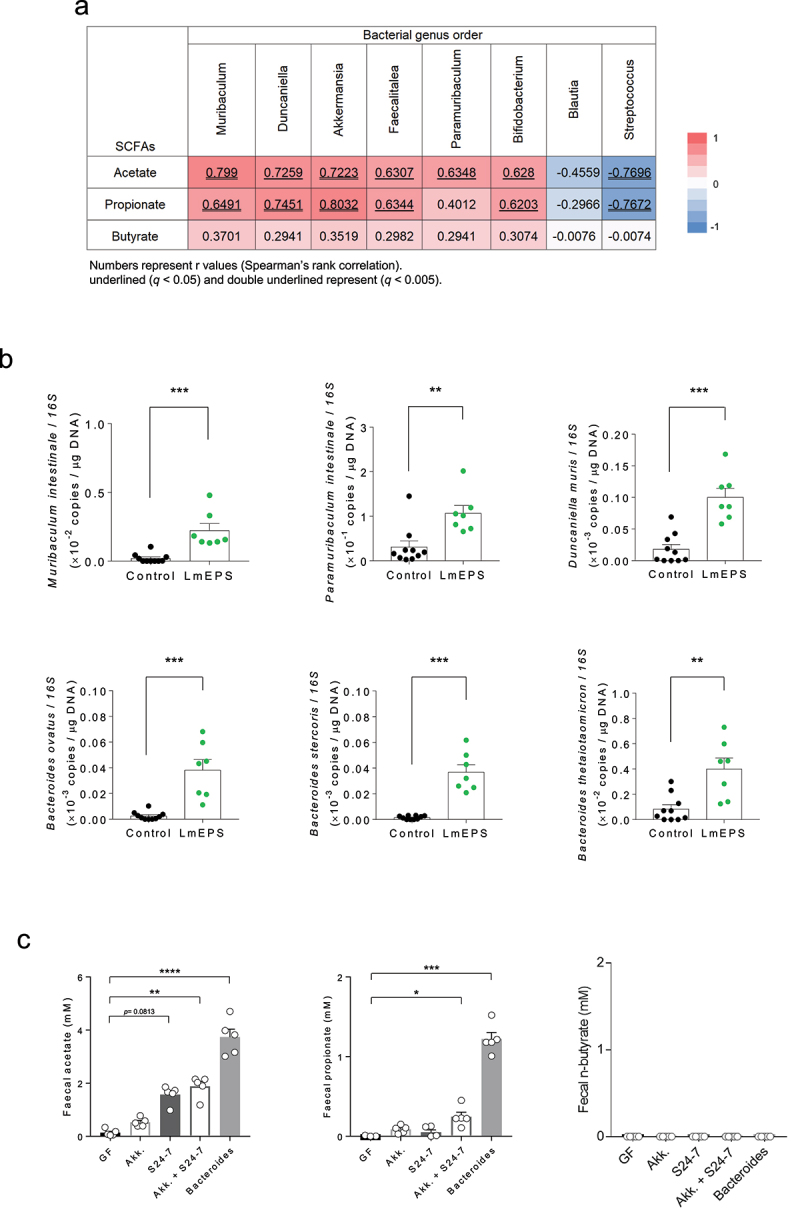
*Leuconostoc mesenteroides*-produced exopolysaccharide (LmEPS) was fermented by *Bacteroidales* S24-7 group and *Bacteroides* in the intestine. (a) Spearman’s rank correlation between the main contributing bacterial genera level and fecal SCFAs in high-fat diet (HFD)-fed cellulose-supplemented (control) mice versus HFD-fed LmEPS-supplemented mice. (b) LmEPS-utilizing *Bacteroidales* S24-7 group and *Bacteroides* species were detected using quantitative polymerase chain reaction (qPCR, *n* = 10, 7 per group). ***P < .001, **P < .01; Mann–Whitney U test). (c) Germ-free and colonized mice (Akk.; Akkermansia muciniphila, S24-7; Bacteroidales S24-7 group, Akk. + S24-7; Akkermansia muciniphila + Bacteroidales S24-7 group, and Bacteroides; Bacteroides 3 species) were fed an AIN-76A diet containing LmEPS for 2 weeks, after which fecal SCFAs were measured using gas chromatography-mass spectrometry (n = 5). ***P < .001, *P < .05 (Dunn’s post-hoc test). Results are presented as the mean ± standard error of the mean (SE)

We observed high correlation coefficient values for the *Bacteroidales* S24-7 group members, such as *Muribaculum, Paramuribaculum*, and *Duncaniella* (Figure 6a). Additionally, the abundance of *M. intestinale, P. intestinale, D. muris, B. ovatus, B. sterocoris*, and *B. thetaiotaomicron*, which efficiently produced SCFAs during *in vitro* monoculture (Figure 1b), was markedly increased following LmEPS intake (Figure 6b). We then performed a transfer experiment for these species (*Bacteroides* and *Bacteroidales* S24-7 group) and confirmed their intestinal colonization (Supplementary Table 2). Compared to those in GF mice, the fecal acetate and propionate levels in *Bacteroides*-colonized mice were significantly higher following the intake of LmEPS (Figure 6c). Although the *Bacteroidales* S24-7 group did not directly produce propionate, *Akkermansia* may be involved in propionate production via polysaccharide transformation by the *Bacteroidales* S24-7 group.^34,35^ Following the intake of LmEPS, the fecal acetate and propionate levels in the *Bacteroidales* S24-7 group and *Akkermansia*-co-colonized mice were significantly higher than those in GF mice (Figure 6c). Among the SCFAs, propionate exerts host metabolic benefits via both GPR41 and GPR43^16^. Thus, propionate, which was produced from LmEPS by gut microbes, is important for the host metabolic benefits elicited by LmEPS.

## Discussion

Consumption of fermented food provides various health benefits to humans, including reduced obesity and allergy.^1^ Although these effects are known to be caused by bacterial functions, the accurate mechanism remains unclear. In this study, we showed that LmEPS affects host metabolic functions as prebiotics via the gut microbiota. However, the metabolic beneficial effects of administering *L. mesenteroides* (10^9^ cfu/g) were considerably weaker than those conferred by LmEPS administration.

*L. mesenteroides* can produce EPS in the presence of sucrose but not glucose and fructose.^7,8^ Hence, the amount of sucrose present during the fermentation process may affect EPS production, and different amounts may thus exert different metabolic benefits. Moreover, the structure of EPS in terms of the molecular weight of the main chain length and number of branching carbohydrates may be strain specific.^36,37^ These aspects could influence SCFA production by gut microbes. Additionally, bacterial species other than *L. mesenteroides* also produce various EPS,^38^ and their EPS may affect human health through prebiotic effects. However, *L. mesenteroides* is the microbial species that produces the largest amount of EPS.^39^

EPS intake plays a crucial role in host beneficial metabolic functions as gut microbial fermentation of EPS leads to the production of SCFAs, which exert beneficial effects via SCFA receptors. EPS can be highly varied in terms of the polysaccharide structure, monosaccharide constituents, main chain length, and branching.^40,41^ In this study, LmEPS was metabolized to SCFAs by gut microbes. Nonetheless, EPS itself may directly influence host physiological functions, and SCFAs may affect the host in a receptor-independent manner, such as through histone deacetylase inhibition and *de novo* metabolic function via SCFA transporters,^42,43^ However, the improvement of glucose homeostasis was not confirmed in our experiment using LmEPS-administered GF mice.

Our study showed that promotion of GLP-1 and insulin secretion by LmEPS was suppressed in *Gpr43^–/^^–^* but not in *Gpr41^–/^^–^* mice. Whether GPR41 directly promotes GLP-1 secretion remains unknown.^16^ In addition, GPR41 is mainly activated by propionate and butyrate, whereas GPR43 is activated by acetate and propionate.^16^ LmEPS administration and intake increased acetate and propionate but not butyrate levels in plasma, cecum, and feces. Therefore, LmEPS-induced glucose metabolism may have shifted to GPR43 functions.

Additionally, each of the three species of *Bacteroides* and the *Bacteroidales* S24-7 group efficiently produced SCFAs during *in vitro* monoculture for gut microbial screening. However, although these *Bacteroides* species were not dominant in mice, acetate and propionate levels in *Bacteroides*-colonized mice were markedly higher than those in *Bacteroidales* S24-7 group-colonized mice following the intake of LmEPS. This suggests that intake of LmEPS further increases intestinal acetate and propionate as these species of *Bacteroides* were dominant in humans. To clarify this mechanism, analyses including human participants should be performed in the future. Thus, EPS, as a fermentable dietary fiber, may affect host health through various mechanisms when used as a prebiotic.

In contrast, administration of *L. mesenteroides* exerted subtle metabolic beneficial effects. Considering that LmEPS production is conditioned by the sucrose substrate, the probiotic efficacy of *L. mesenteroides* is likely dependent on high sucrose diets. Future studies including groups of both HFD- and high-fat/high-sucrose diet-fed mice may reveal whether the probiotic efficacy of *L. mesenteroides* is diet dependent.

**Figure 7.**
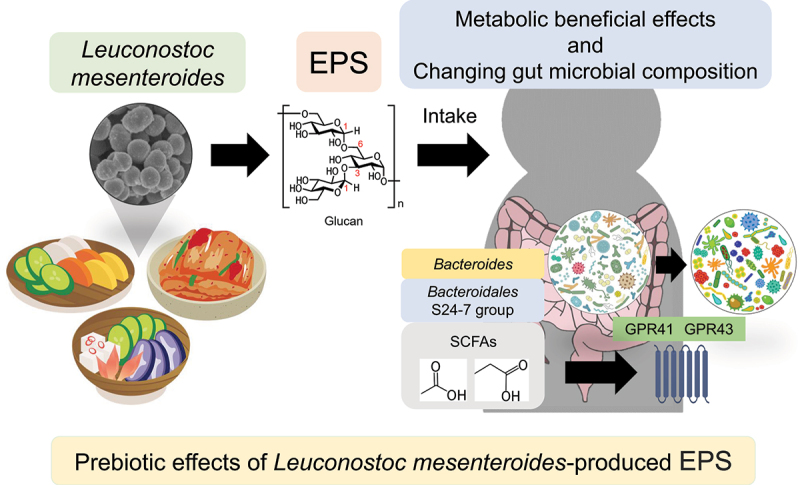
Schematic representation of the prebiotic effects of *Leuconostoc mesenteroides*-produced EPS. *Leuconostoc mesenteroides* produces exopolysaccharides (EPS) in fermented foods. Intake of *Leuconostoc mesenteroides*-produced EPS (LmEPS) changes gut microbial composition. Short-chain fatty acids (SCFAs), which are produced from LmEPS by *Bacteroides* and *Bacteroidales* S24-7 group, exert host metabolic beneficial effects through G-protein coupled receptor GPR41 and GPR43 activation. Thus, intake of fermented foods may exert prebiotic effects via LmEPS.

In the present study, we demonstrated that bacterial EPS may be the primary factor that exerts metabolic benefits through intake of fermented foods. LmEPS, an indigestible polysaccharide, was utilized to produce SCFAs via gut microbial fermentation and changed gut microbial composition. SCFAs produced from LmEPS conferred beneficial metabolic effects to mice. Much evidence revealed that fermentable dietary fibers are crucial for regulating the host gut environment and homeostasis by modulating the gut microbiota (Figure 7). These findings not only suggest a representative central mechanism underlying an interplay between the host and commensal bacteria for energy homeostasis via dietary fibers, but also contribute to the development of functional foods by tailoring the use of EPS for the prevention of lifestyle-related diseases.

## Supplementary Material

Supplemental Material

## Figures and Tables

**Table:** 

DDBJ	DNA Data Bank of Japan
Epi	epididymal
EPS	exopolysaccharide
GF	germ-free
GLP-1	glucagon like peptide-1
GPR	G-protein coupled receptor
GTT	glucose tolerance test
HFD	high fat diet
ITT	insulin tolerance test
JCM	Japan Collection of Microorganisms
LFD	low-fiber diet
LmEPS	*Leuconostoc mesenteroides*-produced EPS
MRS	de Man, Rogosa, and Sharpe
NEFA	non-esterified fatty acids
OGTT	oral glucose tolerance test
Peri	perirenal
PYY	peptide YY
SCFA	short-chain fatty acid
Sub	subcutaneous
TG	triglyceride
WAT	white adipose tissue

## Data Availability

A reporting summary for this Article is available as a Supplementary Information file. The source data for Figures 1–6, Supplementary Figures 1–5, and Supplementary Table 2 are provided as a Source Data file. The draft genome sequence of *L. mesenteroides* has been deposited in the whole genome database of the DNA Data Bank of Japan (DDBJ) under Bio Project Accession No. BOPR01000001. The 16S rRNA sequencing data have been deposited into the DDBJ under the accession No. DRA013084 and DRA013085. All other data generated or analyzed during this study are included in this published article, and its Supplementary Information files or are available from the corresponding authors upon reasonable request.
