# Supplementary material for: Host metabolic benefits of prebiotic exopolysaccharides produced by Leuconostoc mesenteroides
Source: Gut Microbes. 2023 Jan 5;15(1):2161271. doi: 10.1080/19490976.2022.2161271 (PMC9828693; doi:10.1080/19490976.2022.2161271)
Supplement: Supplemental Material [file KGMI_A_2161271_SM5330.docx]

**Host metabolic benefits of prebiotic exopolysaccharides produced by *Leuconostoc mesenteroides***

Junki Miyamoto^a,b#^, Hidenori Shimizu^b,c#^, Keiko Hisa^b,c^, Chiaki Matsuzaki^d^, Shinsuke Inuki^b,e^, Yuna Ando^f^, Akari Nishida^g^, Ayano Izumi^g^, Mayu Yamano^g^, Chihiro Ushiroda^a,h^, Junichiro Irie^b,i^, Takane Katayama^j^, Hiroaki Ohno^b,e^, Hiroshi Itoh^b,i^, Kenji Yamamoto^k^, Ikuo Kimura^a,b,f,g*^

^a^Department of Applied Biological Science, Graduate School of Agriculture, Tokyo University of Agriculture and Technology, Fuchu-shi, Tokyo 183-8509, Japan, ^b^AMED-CREST, Japan Agency for Medical Research and Development, Chiyoda-ku, Tokyo 100-0004, Japan, ^c^Noster Inc., Kamiueno, Muko, Kyoto 617-0006, Japan, ^d^Research Institute for Bioresources and Biotechnology, Ishikawa Prefectural University, Ishikawa 921-8836, Japan, ^e^Department of Bioorganic Medicinal Chemistry and Chemogenomics, Graduate School of Pharmaceutical Sciences, Kyoto University, Sakyo-ku, Kyoto 606-8501, Japan, ^f^Laboratory of Molecular Neurobiology, Graduate School of Biostudies, Kyoto University, Sakyo-ku, Kyoto 606-8501, Japan, ^g^Department of Molecular Neurobiology, Graduate School of Pharmaceutical Sciences, Kyoto University, Kyoto 606-8501, Japan, ^h^Department of Clinical Nutrition, Fujita Health University, Aichi 470-1101, Japan, ^i^Department of Endocrinology, Metabolism and Nephrology, School of Medicine, Keio University, Shinjuku-ku, Tokyo 160-8582, Japan., ^j^Laboratory of Molecular Biology and Bioresponse, Graduate School of Biostudies, Kyoto University, Sakyo-ku, Kyoto 606-8501, Japan, ^k^Center for Innovative and Joint Research, Wakayama University, Wakayama, 640-8510, Japan.

^#^J.M. and H.S. contributed equally to this work.

^*^Corresponding author: Ikuo Kimura

Email: [kimura.ikuo.7x@kyoto-u.ac.jp](mailto:kimura.ikuo.7x@kyoto-u.ac.jp)


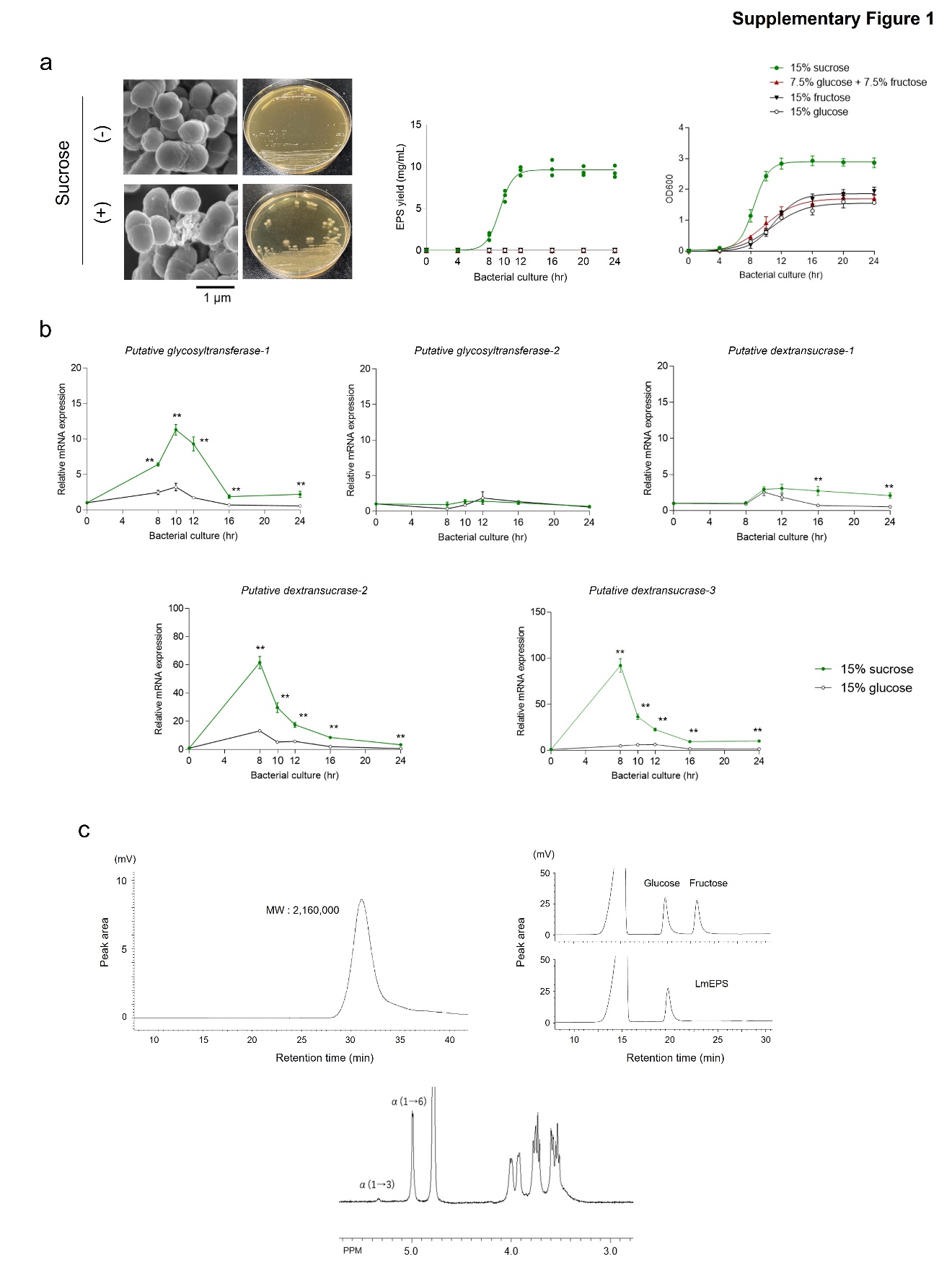


**Supplementary Figure 1. Characterization and construction of *Leuconostoc mesenteroides*-produced exopolysaccharide** (**LmEPS).** (**a**) Scanning electron micrograph of colony and LmEPS biosynthesis (scale bars, 1 μm). Growth curves of EPS biosynthesis and optical density at 600 nm (OD_600_) (*n* = 3 per group). (**b**) Expression of putative glycosyltransferase and dextransucrase mRNAs of *Leuconostoc mesenteroides* in MRS medium containing sucrose or glucose was monitored (*n* = 6 per group). ***P* < 0.01, **P* < 0.05 (Mann–Whitney U test). (**c**) Molecular mass distribution and monosaccharide composition were measured using high-pressure liquid chromatography (HPLC), and structural characterization was performed using proton nuclear magnetic resonance (^1^H NMR) spectroscopy. Results are presented as the mean ± standard error of the mean (SE).


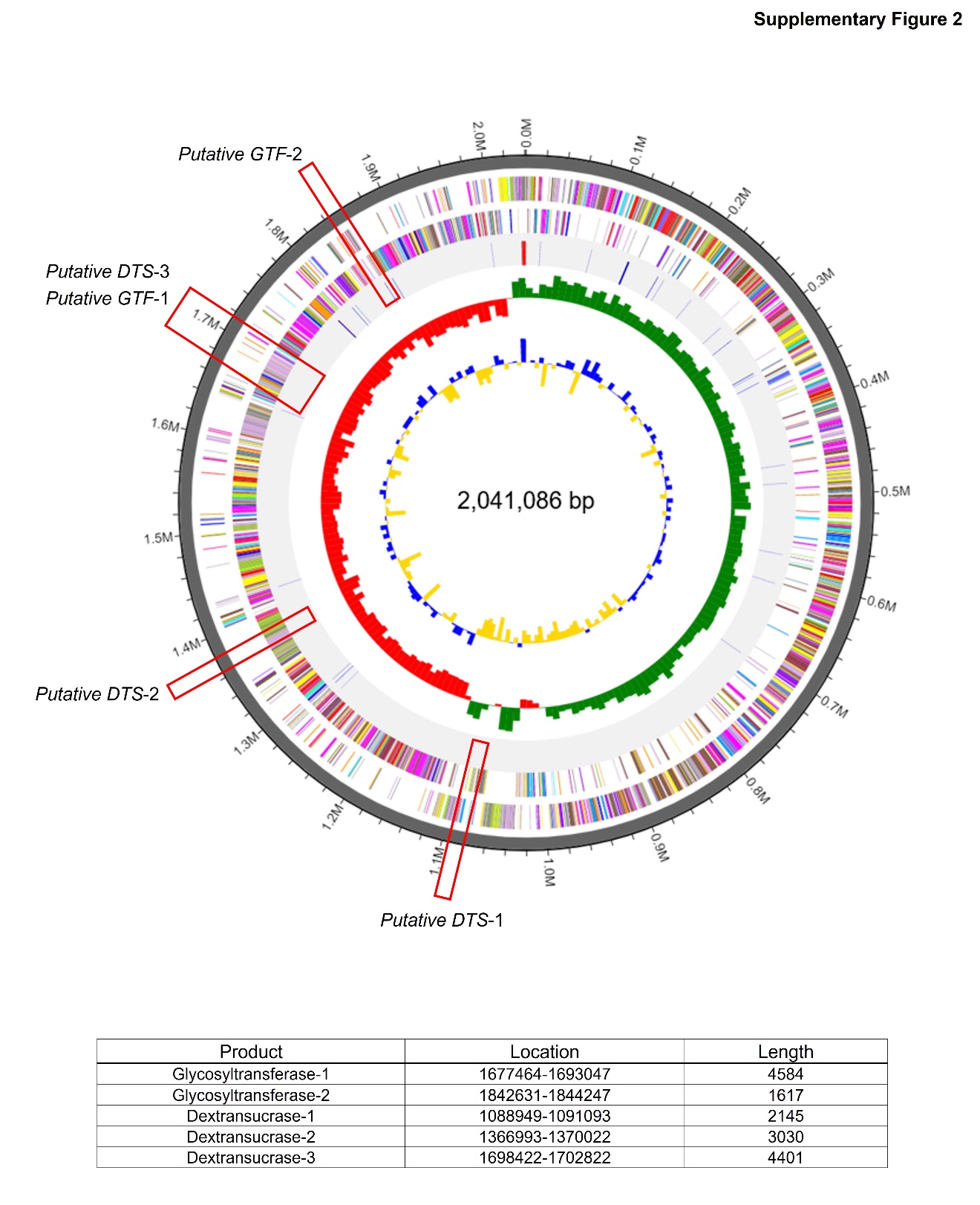


**Supplementary Figure 2. Two putative glycosyltransferase and three putative dextransucrase genes from the draft genome sequence of *Leuconostoc mesenteroides* NTM048.**

**
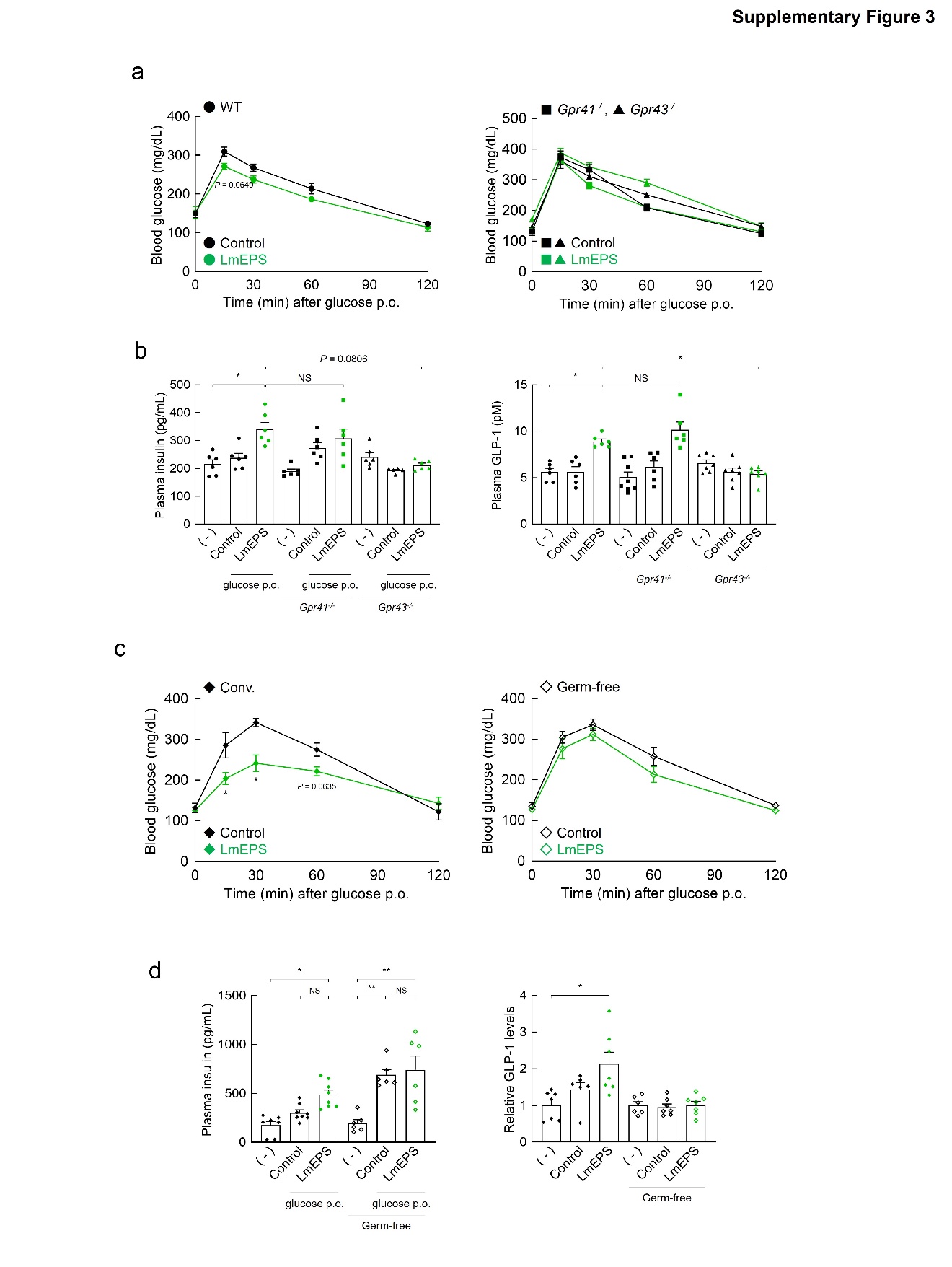
**

**Supplementary Figure 3. Effects of *Leuconostoc mesenteroides*-produced exopolysaccharide** (**LmEPS) on glucose homeostasis of mice.** (**a**) After 24-h fasting, each mouse was fed a 0.2 g AIN-76A diet containing 50% cellulose (control) or 50% LmEPS, and oral glucose tolerance test was performed 1 h after feeding. C57BL/6 (*n* = 7 per group), *Gpr41^–/–^* (*n* = 8 per group), and *Gpr43^–/–^* mice (*n* = 7, 8 per group) were used. (**b**) The plasma insulin and glucagon-like peptide-1 (GLP-1) levels were measured 30 min after glucose administration. C57BL/6 (*n* = 6 per group), *Gpr41^–/–^* (*n* = 6 per group for insulin and *n* = 8, 6, 6 per group for GLP-1), *Gpr43^–/–^* mice (*n* = 6, 5, 6 per group for insulin and *n* = 7 per group for GLP-1). (**c**) After 24-h fasting, each mouse was fed a 0.2 g AIN-76A diet containing 50% cellulose (control) or 50% LmEPS, and an oral glucose tolerance test was performed 1 h after feeding. ICR (*n* = 5, 6 per group) and germ-free (GF)-ICR mice (*n* = 5 per group). (**d**) The plasma insulin and GLP-1 levels were measured 30 min after glucose administration. ICR (*n* = 7, 8, 8 per group for insulin and *n* = 7, 6, 7 per group for GLP-1) and GF-ICR mice (*n* = 6 per group for insulin and *n* = 6, 7, 7 per group for GLP-1) were used. ***P* < 0.01, **P* < 0.05 (Mann–Whitney U test and Dunn's post-hoc test). Results are presented as means ± standard error of the mean (SE). NS; not significant.


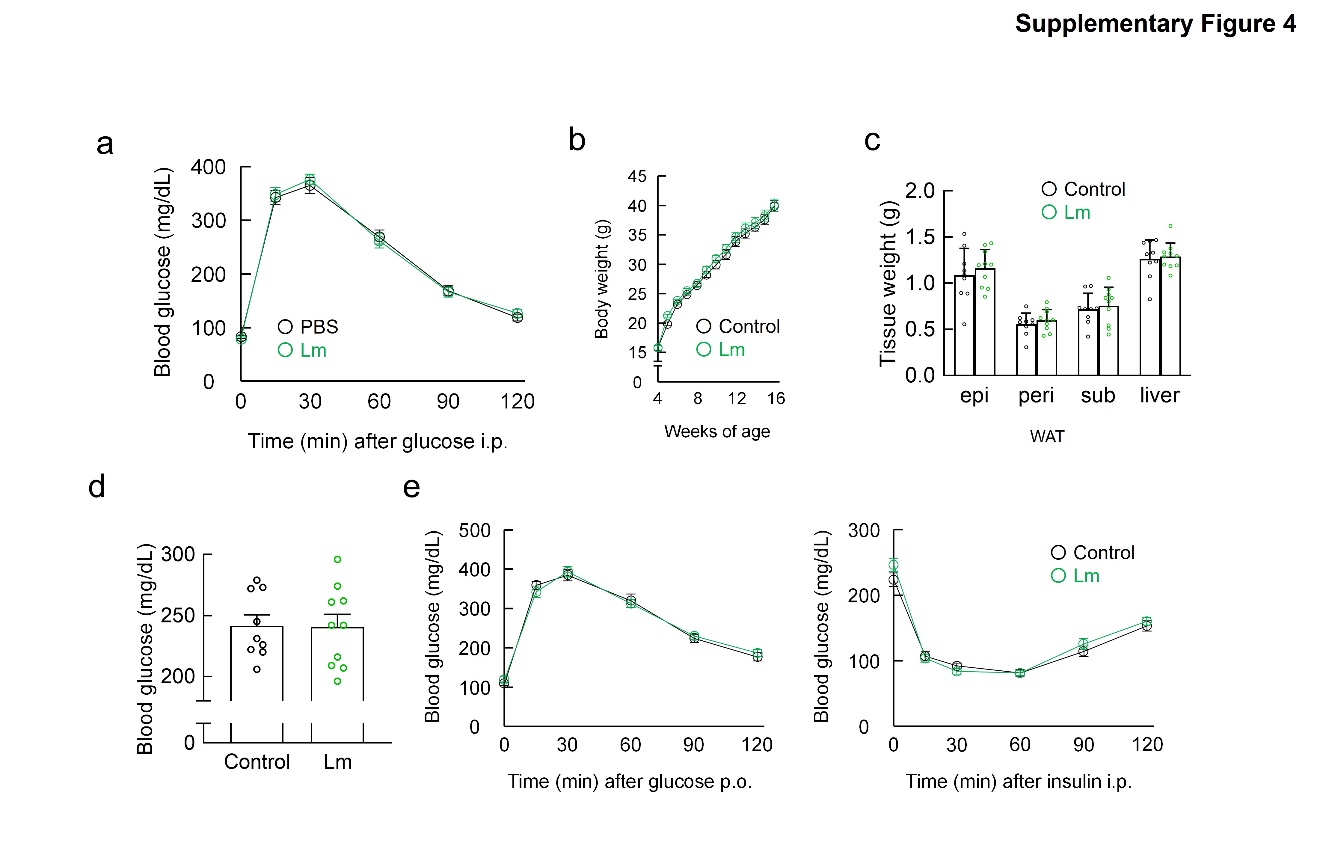


**Supplementary Figure 4. Probiotic effects by *Leuconostoc mesenteroides* on metabolic functions.** (a) After 24-h fasting, the mice (*n* = 8 per group) were administered PBS or *L. mesenteroides* (1 × 10^9^ cfu/mouse) via oral gavage. Intraperitoneal glucose tolerance test was performed 1 h after oral administration. (b–e) Mice were fed a high-fat diet (HFD; control) and *L. mesenteroides*-containing HFD (Lm, 1 × 10^9^ cfu/g) for 12 weeks. (b) Changes in body weights (*n* = 9, 10 per group). (c) Changes in tissue weights (*n* = 9, 10 per group). epi, epididymal; peri, perirenal; sub, subcutaneous. WAT; white adipose tissue. (d) Blood glucose was measured at the end of the experimental period (*n* = 9, 10 per group). (e) Oral glucose tolerance test (*Left*, *n* = 9, 10 per group) and insulin tolerance test (*Right*, *n* = 9, 10 per group) were performed at 13–14 weeks of age. Results are presented as the mean ±  standard error of the mean (SE).


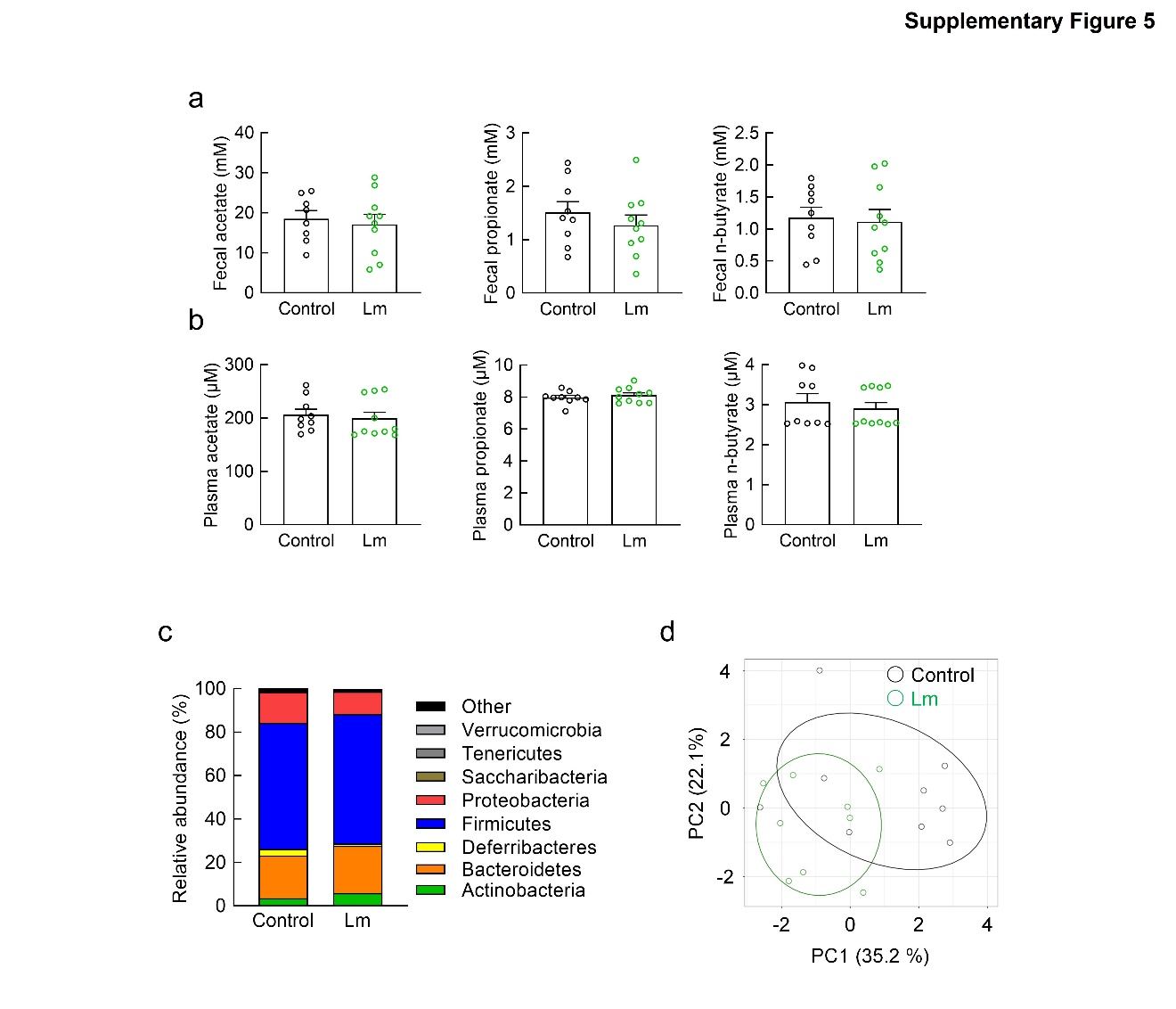


**Supplementary Figure 5. Probiotic effects of *Leuconostoc mesenteroides* on intestinal environments.** (a) Fecal (*n* = 8, 10 per group) and (b) plasma (*n* = 9, 10 per group) short-chain fatty acids (SCFAs) were measured using gas chromatography-mass spectrometry. Results are presented as the mean ± standard error of the mean (SE). (c and d) Gut microbial composition was evaluated to determine the relative abundance of microbial phylum (c) and principal coordinates analysis (d) (*n* = 9 per group).

**Supplementary Table 1. Composition of high-fat diets for *Leuconostoc mesenteroides*-produced exopolysaccharide (LmEPS) supplementation experiments.**


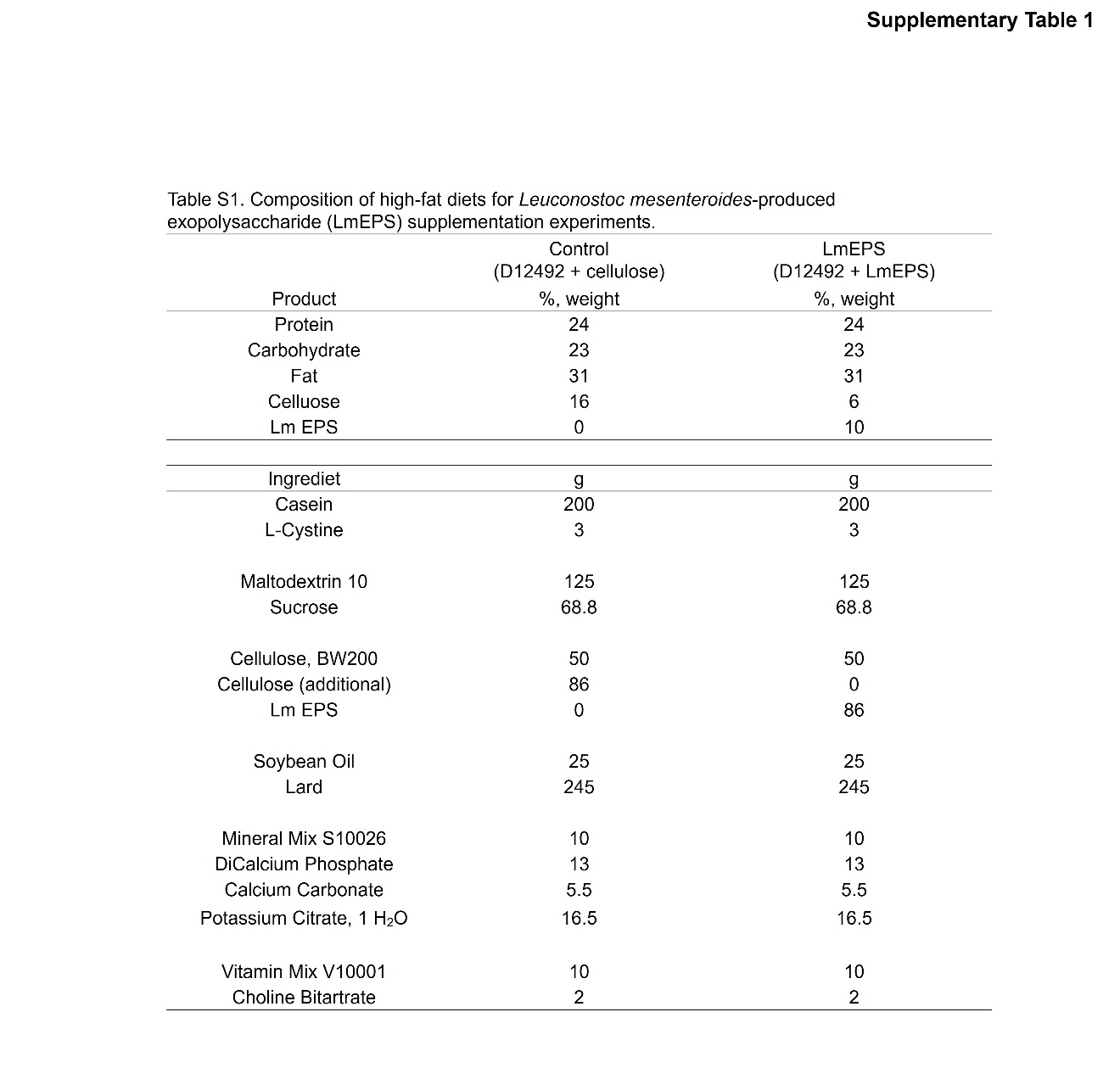


**Supplementary Table 2. Bacterial colonization. Each bacterial colony was detected by qPCR (*n* = 5 samples per group).**


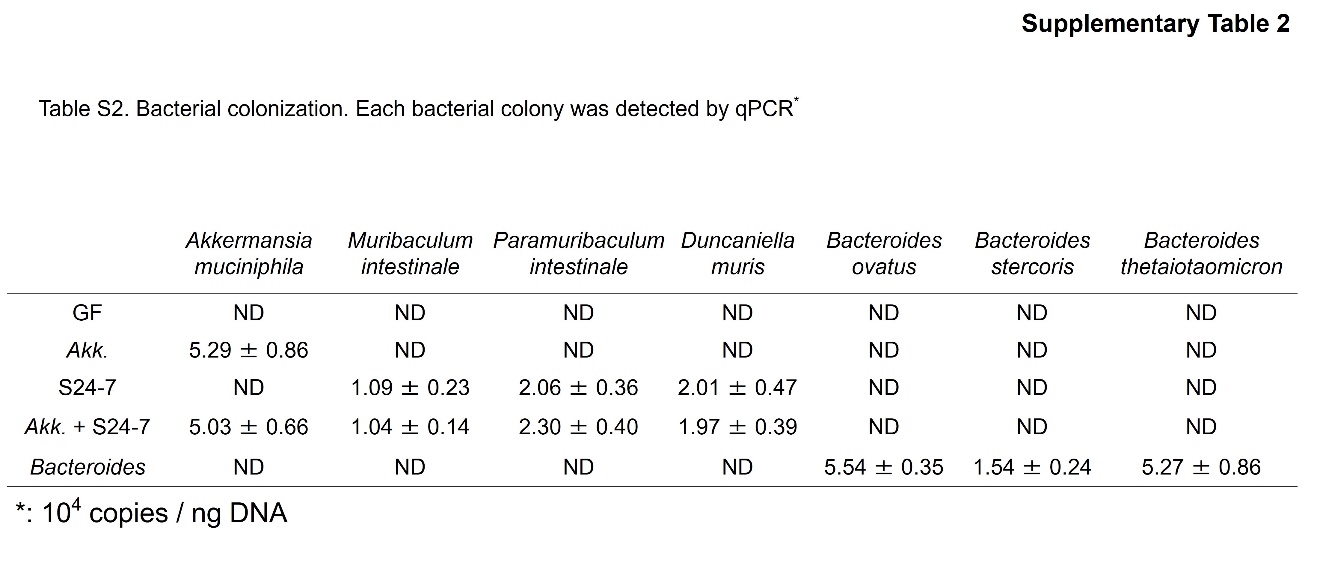


**Supplementary Table 3. Composition of diets for *Leuconostoc mesenteroides*-produced exopolysaccharide (LmEPS) supplementation experiments with AIN-76A.**

**
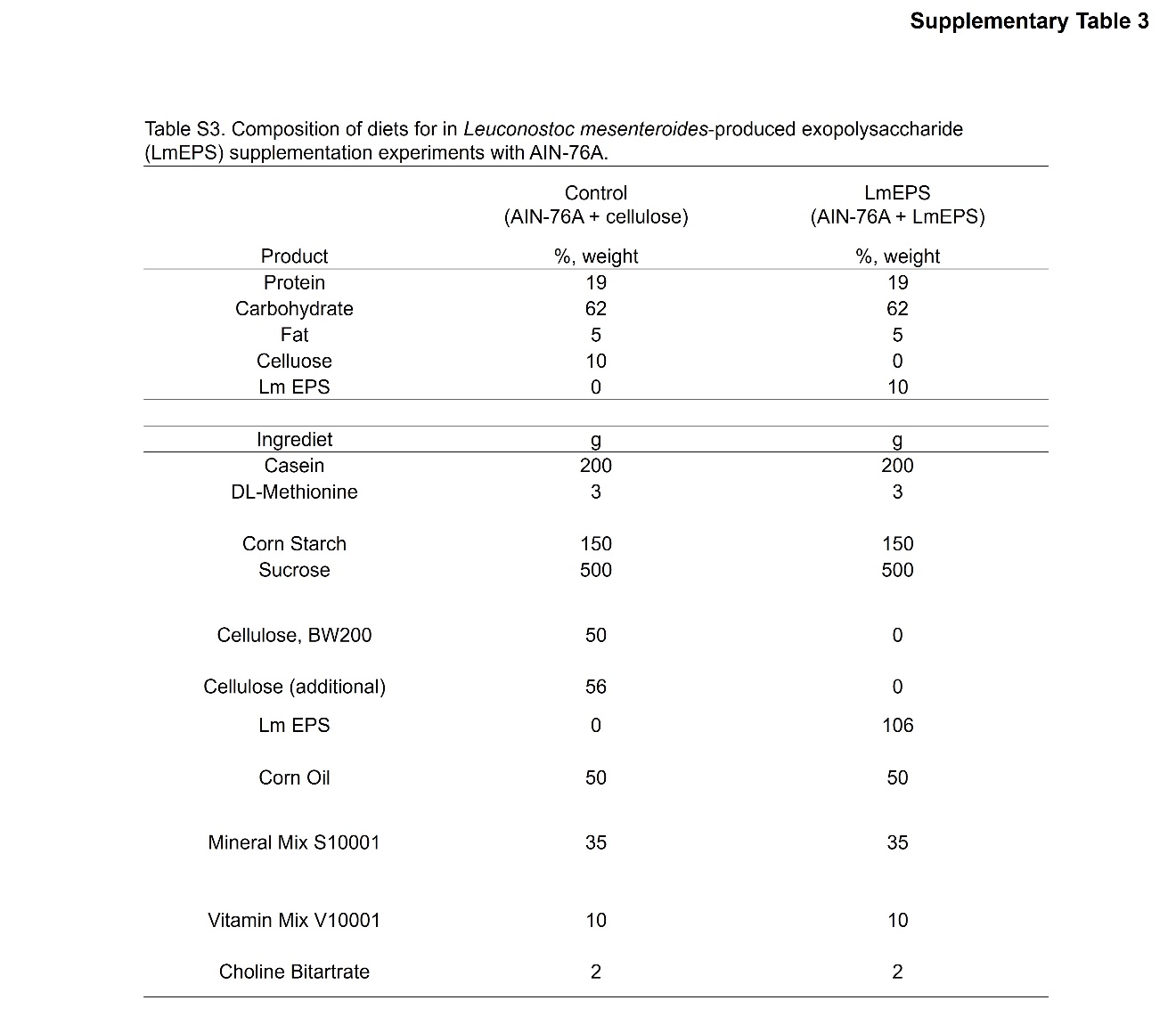
**
